# Deletion of Hypoxia-Inducible Factor-1α in Adipocytes Enhances Glucagon-Like Peptide-1 Secretion and Reduces Adipose Tissue Inflammation

**DOI:** 10.1371/journal.pone.0093856

**Published:** 2014-04-04

**Authors:** Yoshitaka Kihira, Mariko Miyake, Manami Hirata, Yoji Hoshina, Kana Kato, Hitoshi Shirakawa, Hiroshi Sakaue, Noriko Yamano, Yuki Izawa-Ishizawa, Keisuke Ishizawa, Yasumasa Ikeda, Koichiro Tsuchiya, Toshiaki Tamaki, Shuhei Tomita

**Affiliations:** 1 Department of Pharmacology, Institute of Health Biosciences, The University of Tokushima Graduate School, Tokushima, Japan; 2 Department of Medical Pharmacology, Institute of Health Biosciences, The University of Tokushima Graduate School, Tokushima, Japan; 3 Student Laboratory, Faculty of Medicine, The University of Tokushima, Tokushima, Japan; 4 Laboratory of Nutrition, Graduate School of Agricultural Science, Tohoku University, Miyagi, Japan; 5 Department of Nutrition and Metabolism, Institute of Health Biosciences, The University of Tokushima Graduate School, Tokushima, Japan; 6 Division of Molecular Pharmacology, Faculty of Medicine, Tottori University, Tottori, Japan; University of Leicester, United Kingdom

## Abstract

It is known that obese adipose tissues are hypoxic and express hypoxia-inducible factor (HIF)-1α. Although some studies have shown that the expression of HIF-1α in adipocytes induces glucose intolerance, the mechanisms are still not clear. In this study, we examined its effects on the development of type 2 diabetes by using adipocyte-specific HIF-1α knockout (ahKO) mice. ahKO mice showed improved glucose tolerance compared with wild type (WT) mice. Macrophage infiltration and mRNA levels of monocyte chemotactic protein-1 (MCP-1) and tumor necrosis factor α (TNFα) were decreased in the epididymal adipose tissues of high fat diet induced obese ahKO mice. The results indicated that the obesity-induced adipose tissue inflammation was suppressed in ahKO mice. In addition, in the ahKO mice, serum insulin levels were increased under the free-feeding but not the fasting condition, indicating that postprandial insulin secretion was enhanced. Serum glucagon-like peptide-1 (GLP-1) levels were also increased in the ahKO mice. Interestingly, adiponectin, whose serum levels were increased in the obese ahKO mice compared with the obese WT mice, stimulated GLP-1 secretion from cultured intestinal L cells. Therefore, insulin secretion may have been enhanced through the adiponectin-GLP-1 pathway in the ahKO mice. Our results suggest that the deletion of HIF-1α in adipocytes improves glucose tolerance by enhancing insulin secretion through the GLP-1 pathway and by reducing macrophage infiltration and inflammation in adipose tissue.

## Introduction

Type 2 diabetes occurs frequently in obese humans compared with lean individuals. Two features characterize obesity-induced type 2 diabetes. One is the failure of insulin secretion by pancreatic β-cells and the other is insulin resistance [1], [2].

Insulin secretion deficiency is caused by pancreatic β-cell dysfunction and apoptosis [3], [4]. In addition, incretin hormones contribute to the deficiency. Glucagon-like peptide-1 (GLP-1), a major incretin hormone, is released from intestinal L cells in response to nutrients and stimulates pancreatic insulin secretion. It is known that in patients with type 2 diabetes, GLP-1 secretion is diminished [5], [6]. Therefore, it is considered that the diminished GLP-1 secretion is a cause of decreased insulin secretion.

Insulin resistance is the other cause of type 2 diabetes. In obese adipose tissues, adipocytes accumulate excess fat and become hypertrophic, causing aberrant metabolism and abnormal adipokine secretion. Characteristically, the hypertrophied adipocytes secrete monocyte chemotactic protein-1 (MCP-1) [7], which enhances macrophage infiltration into the adipose tissues [8]–[10]. The infiltration of macrophages initiates low-grade inflammation in the adipose tissues, producing inflammatory cytokines, such as tumor necrosis factor α (TNFα) [11]–[13], which in turn cause adipocyte dysfunction [14]–[17]. TNFα causes insulin resistance by directly acting on muscle insulin signaling [18]. In addition, adiponectin, whose serum levels are inversely related to insulin resistance, is reduced in obese adipose tissues [19]–[21]. Such abnormal adipokine secretion causes whole-body insulin resistance.

It is widely suggested that adipose tissues are poorly oxygenated in obese humans and mice, resulting in the induction of hypoxia-inducible factor (HIF)-1α [22]–[26]. Previous studies have examined the involvement of HIF-1α in the development of obesity-induced diabetes by using genetically modified mice [26]–[28]. The expression of a constitutively active form of HIF-1α in adipocytes conferred glucose intolerance [26] and the disruption of HIF-1α in adipocytes improved glucose tolerance [28], indicating that HIF-1α expression in adipocytes deteriorates glucose tolerance. However, it was also reported that the inhibition of HIF-1α in adipocytes together with the expression of a dominant negative form of HIF-1α induced glucose intolerance [27]. Therefore, the effects of HIF-1α on the progression of diabetes remain controversial.

In the present study, we prepared adipocyte-specific HIF-1α knockout (ahKO) mice and studied the effects of HIF-1α on the development of obesity-induced diabetes. Our results suggest that its deletion in adipocytes leads to the improvement of glucose tolerance via two routes: enhancement of insulin secretion through the adiponectin-GLP-1 pathway and reduction of inflammation by decreasing MCP-1 expression.

## Materials and Methods

### Ethics Statement

This study was carried out in strict accordance with the guidelines of the Animal Research Committee of the University of Tokushima, and protocols were approved by the Animal Research Committee of the University of Tokushima (Permit Number: Toku-Doubutsu 11129).

### Antibodies

Anti-F4/80 antibody (AbD Serotec), anti-alpha 1 sodium potassium (NaK) ATPase antibody (Abcam), anti-HIF-1α antibody (Cayman Chemical), anti-HIF-2α antibody (Novus Biologicals), anti-HIF-1β antibody (Novus Biologicals), anti-α-tubulin antibody (Sigma), anti-insulin antibody (Cell Signaling), and anti-glucagon antibody (Abcam) were used.

### HIF-1α conditional knockout mice

Hif1α-floxed (Hif1αF/F) mice containing loxP sites flanking exons 13–15 of the Hif1α gene [29] were crossed with mice harboring the Cre recombinase under the control of the aP2 promoter (aP2-Cre mice; a gift from Ronald M. Evans, Salk Institute for Biological Studies, CA) and under the control of the lysozyme M promoter (LysM-Cre mice; a gift from Irmgard Förster, Institut für Umweltmedizinische Forschung, Heinrich-Heine-Universität Düsseldorf, Düsseldorf, Germany [30]), generating adipocyte-specific HIF-1α knockout (ahKO) mice and macrophage-specific HIF-1α knockout (mhKO) mice, respectively. All mice were on the C57BL/6 background and only male mice were used for experiments. The mice were maintained under temperature- and light-controlled environmental settings with free access to water and dissection was performed in the morning. Six-week-old mice were fed either a normal diet (ND) (10% kcal consisting of fat; MF (Oriental Yeast)) or a high fat diet (HFD) (57% kcal consisting of fat; high fat diet 32 (CLEA Japan)) for another 20 weeks.

### Glucose and insulin tolerance tests

The glucose tolerance test (GTT) was performed in mice at the age of 15 weeks. Two grams of glucose per kg body weight was administered orally (OGTT) or intraperitoneally (ipGTT). In some experiments, 25 μg of exendin (9–39) (Ex9–39; Sigma) per kg body weight was injected 15 min before glucose administration. The insulin tolerance test (ITT) was performed by intraperitoneally administering 1 U of recombinant human insulin (Eli Lilly Japan) per kg body weight. After injection of glucose or insulin, blood glucose levels were monitored from the tail vein by using a G-Checker Self-Monitoring Blood Glucose System (Gunze).

### Western blot

An epididymal fat pad was lysed in lysis buffer (20 mM Tris-HCl (pH 8.0), 0.15 M NaCl, 1 mM phenylmethylsulfonyl fluoride, 1% Triton X-100, protease inhibitor mixture (2 g/ml aprotinin, 1 μg/ml leupeptin, 2 μg/ml antipain, and 10 μg/ml benzamidine), and phosphatase inhibitor mixture (10 mM NaF, 60 mM β-glycerophosphate, 10 mM sodium pyrophosphate, 2 mM sodium orthovanadate)). Proteins were separated on SDS polyacrylamide gels and electrophoretically transferred to polyvinylidene fluoride membranes. The membranes were incubated with different primary antibodies overnight at 4°C and then probed with an HRP-conjugated secondary antibody (KPL). Immunoreactive bands were detected with ECL (GE Healthcare) and visualized by exposing the membranes to X-ray films (GE Healthcare). The proteins were quantified by densitometric analysis using ImageJ analysis software.

### Immunohistochemical analyses

For immunofluorescence staining of adipose tissues, epididymal adipose tissues were fixed with 4% paraformaldehyde, after which epididymal fat was removed and minced into small pieces (∼2 to 3 mm). The tissue pieces were boiled in 10 mM citrate buffer for 15 min and treated with 0.3% Triton X-100 in Tris-buffered saline (TBS; 150 mM NaCl and 20 mM Tris-HCl (pH 8.0)) for 10 min. Then, the tissue pieces were blocked with TBS containing 10% normal goat serum for 1 h at room temperature. In some experiments, Hoechst 33342 (Dojindo) and BODIPY (Molecular Probes) were added to the blocking solution. The tissue pieces were incubated with a primary antibody overnight at 4°C and then washed and probed with Alexa-conjugated secondary reagents. For immunofluorescence staining of pancreas, paraffin sections of pancreas were deparaffinized and rehydrated. The sections were boiled in 10 mM citrate buffer for 15 min and treated with 0.3% Triton X-100 in TBS for 10 min. Then, the sections were blocked with TBS containing 10% normal goat serum for 1 h at room temperature. Thereafter, sections were incubated with blocking solution containing primary antibodies and Hoechst 33342 overnight at 4°C. Finally, the sections were washed and probed with Alexa-conjugated secondary reagents. Fluorescent images were taken with an A1 confocal microscope configured with a Ti-E inverted microscope (Nikon). The number of crown-like structures (CLS) was determined by counting the total number of CLS in a 1 mm^2^ visual field.

### Measurement of serum concentrations of hormones

To analyze serum, we conducted insulin ELISA (Morinaga), GLP-1 (Active) ELISA (Shibayagi), GIP ELISA (Millipore), and adiponectin ELISA (Otsuka). To detect adipokines, namely, leptin, tissue plasminogen activator inhibitor-1 (tPAI-1), resistin, and MCP-1, a MILLIPLEX MAP Mouse Adipokine Magnetic Bead Panel (Millipore) was used.

### Real-time PCR

Total RNA was isolated from epididymal adipose tissue, liver, or femoral muscle with ISOGEN (Nippon Gene). One μg of total RNA was subjected to RT-PCR using a PrimeScript RT-PCR Kit with Oligo dT Primer (Takara). The following primers were used for the analyses: HIF-1α forward (5′-GCAGCAGGAATTGGAACATT-3′), HIF-1α reverse (5′-GCATGCTAAATCGGAGGGTA-3′), HIF-2α forward (5′-CCTGCAGCCTCAGTGTATCA-3′), HIF-2α reverse (5′-GTGTGGCTTGAACAGGGATT-3′), HIF-1β forward (5′-TCTCCCTCCCAGATGATGAC-3′), HIF-1β reverse (5′-CAATGTTGTGTCGGGAGATG-3′), F4/80 forward (5′-CTGTAACCGGATGGCAAACT-3′), F4/80 reverse (5′-CTGTACCCACATGGCTGATG-3′), TNFα forward (5′-CCAGACCCTCACACTCAGATC-3′), TNFα reverse (5′-CACTTGGTGGTTTGCTACGAC-3′), MCP-1 forward (5′-CCCAATGAGTAGGCTGGAGA-3′), and MCP-1 reverse (5′-TCTGGACCCATTCCTTCTTG-3′).

### GLP-1 secretion from intestinal L cells

GLUTag cells [31] were cultured in DMEM supplemented with 10% fetal bovine serum, penicillin, and streptomycin. The cells were plated at 50,000 cells/cm^2^ in 12-well culture plates and allowed to grow for 48 h. The cells were preincubated with KRH buffer (117 mM NaCl, 4.7 mM KCl, 2.5 mM CaCl_2_, 1.2 mM MgSO_4_, 20 mM HEPES, and 0.1% BSA) for 30 min. Then, the cells were incubated with 10 μM forskolin (Fsk)/1-methyl-3-(2-methylpropyl)-7H-purine-2,6-dione (IBMX), 100 nM leptin (Sigma), 10 ng/ml resistin (R&D Systems), or 30 μg/ml adiponectin (recombinant mouse gAcrp30; BioVision) in KRH buffer containing 2.5 mM glucose for 2 h at 37°C. The incubation buffer was collected and centrifuged to remove any floating cells. Then, GLP-1 was subjected to ELISA.

### Statistical studies

The data are expressed as means ± standard error of mean (SEM). Statistical analysis of differences between groups was performed using the paired *t*-test or the one-way analysis of variance followed by Dunnett's or Tukey's multiple comparison tests. Values of *p*<0.05 or *p*<0.01 were statistically significant.

## Results

### Knockout of HIF-1α in adipose tissues

**Figure 1:**
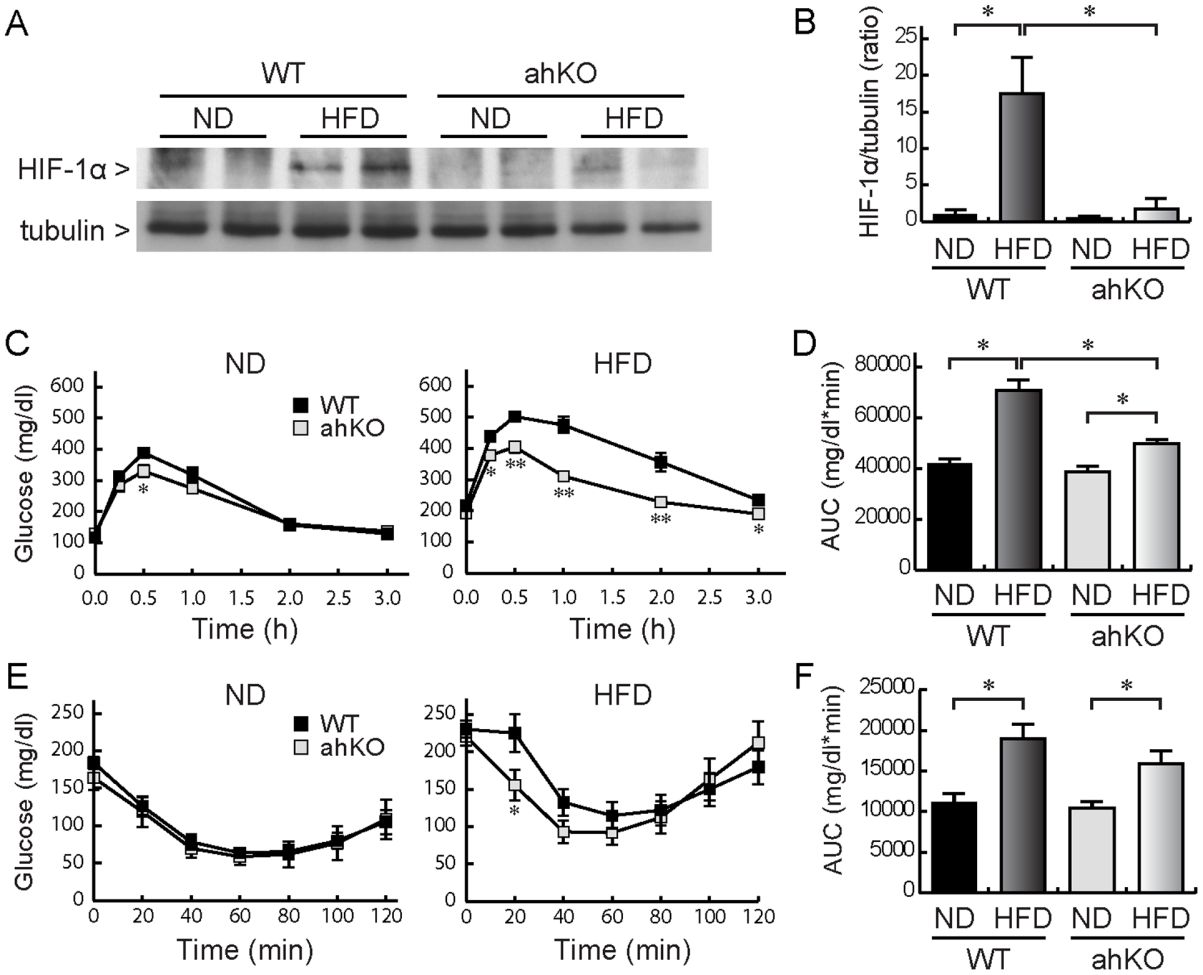
Expression of HIF-1α in epididymal adipose tissue and glucose and insulin tolerance tests. (A) Western blot images of HIF-1α. Epididymal fat pads isolated from ahKO and WT mice were solubilized in lysis buffer and subjected to western blotting. (B) Quantification of protein levels of HIF-1α. Values are means ±S.E.M. (n = 6). *P<0.01. (C) Time-dependent changes of blood glucose concentrations in OGTT. After a 24-h fast, 2 g/kg glucose was injected orally and blood glucose concentrations were measured at the indicated time points. Values are means ±S.E.M. (n = 6∼8). *P<0.05 and **P<0.01. (D) AUCs of glucose concentration in OGTT. Values are means ±S.E.M. (n = 6∼8). *P<0.01. (E) Time-dependent changes of blood glucose concentrations in ITT. After a 4-h fast, 1 U/kg insulin was injected intraperitoneally and blood glucose concentrations were measured. Values are means ±S.E.M. (n = 6∼7). *P<0.05. (F) AUCs of glucose concentration in ITT. Values are means ±S.E.M. (n = 6∼7). *P<0.01.

To determine the effects of HIF-1α in adipocytes on the progression of obesity-induced diabetes, we prepared ahKO mice and recorded changes when the mice were fed a normal diet (ND) or a high-fat diet (HFD). First, we confirmed the expression of the HIF family in epididymal adipose tissues. As shown in Fig. S1A, HIF-1α mRNA expression was significantly reduced in the epididymal adipose tissues of ahKO mice compared with those of wild type (WT) mice under both ND and HFD conditions. Significant decreases in HIF-1α mRNA expression were not observed in liver and muscle, both of which are important organs for glucose metabolism (Fig. S1A). HIF-1α protein expression was increased in epididymal adipose tissues of HFD-induced obese WT mice (hereafter HFD WT mice) (Fig. 1A and 1B). As expected, the increase was abolished in ahKO mice. HIF-2α and HIF-1β expression exhibited no differences between the two conditions (Fig. S1B and S1C), indicating that HIF-1α is specifically knocked out in adipose tissues without compensation by the other HIF family proteins.

### Glucose tolerance and insulin resistance in ahKO mice

As shown in Table 1, HFD WT mice exhibited significant increases in body weight, weights of epididymal and subcutaneous fat pads, adipocyte size, and blood glucose compared with ND WT mice. The changes noted in ahKO mice were nearly identical to those found in WT mice. These results indicated that obesity was equally induced in WT and ahKO mice. However, the oral glucose tolerance test (OGTT) revealed marked differences in glucose clearance between the two mice. As shown in Fig. 1C and 1D, even in the ND condition, serum glucose levels in ahKO mice were significantly decreased at 30 min after glucose injection although the areas under the curves (AUCs) were not significantly different. Under the HFD condition, low glucose levels were observed in ahKO mice from 15 min to 3 h after glucose injection together with a significant decrease of AUC. In addition, insulin resistance was determined with the insulin tolerance test (ITT). No significant differences in the time courses of serum glucose levels were noted between WT and ahKO mice under the ND condition, whereas glucose levels in ahKO mice were significantly lower than those in WT mice at 20 min after the insulin injection under the HFD condition (Fig. 1E and 1F). These results suggested that ahKO mice have improved glucose tolerance and insulin sensitivity.

**Table 1:** Characteristics of ahKO mice.

Category	ND WT	HFD WT	ND ahKO	HFD ahKO
Body weight (g)	32.8±1.4	45.7±1.0*	34.9±5.0	46.5±2.1†
Weight of Epi fat (mg/g b.w.)	21.4±3.0	50.0±4.3*	28.3±4.0	51.1±4.3†
Weight of Sc fat (mg/g b.w.)	18.1±1.5	54.4±4.0*	18.7±2.3	54.2±2.1†
Blood glucose (mg/dl)	112.8±4.2	213.0±8.9*	109.1±17.5	188.1±7.6†
Adipocyte size (μm^2^)	4844.6±607.88	8803.0±796.5*	5383.5±899.7	10383.6±484.3†

Body weights, epididymal (Epi) fat weights, subcutaneous (Sc) fat weights, blood glucose concentrations, and adipocyte sizes of 25-week-old mice are indicated. Values are means ±S.E.M.

*P<0.01 vs. WT-ND mice and †*P*<0.01 vs. ahKO-ND mice. No category is significantly different between WT-ND mice and ahKO-ND mice and between WT-HFD mice and ahKO-HFD mice.

### Inflammation of adipose tissues

**Figure 2:**
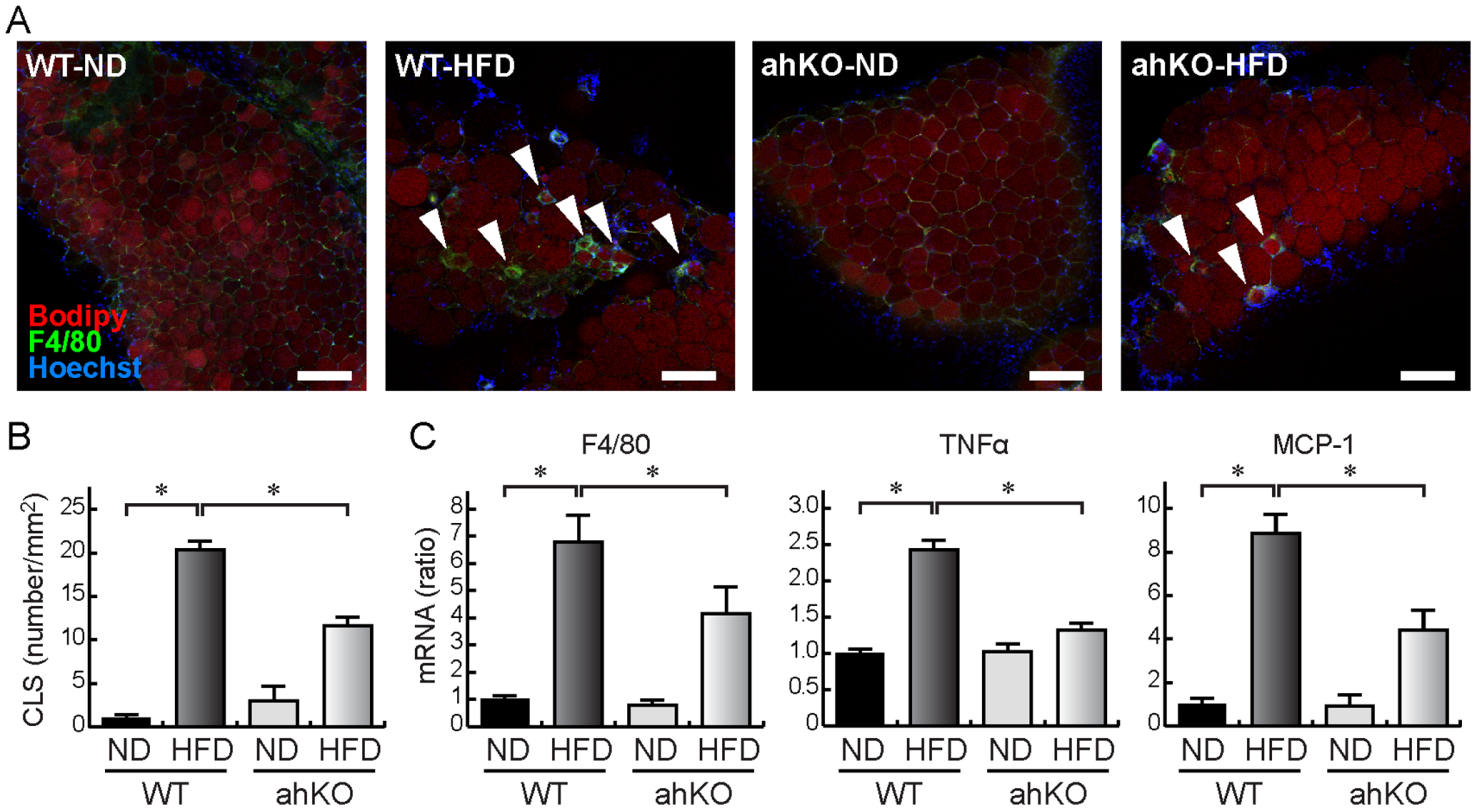
Inflammation of epididymal adipose tissues. (A) Immunostaining for F4/80. Isolated epididymal fat pads were immunostained with anti-F4/80 antibody (green). Adipocytes were counterstained with BODIPY (red) and nuclei, with Hoechst (blue). Bars indicate 200 μm. Arrowheads indicate CLS. (B) The number of CLS in a 1 mm^2^ visual field was counted. Values are means ±S.E.M. (n = 6). *P<0.01. (C) Real-time PCR analyses of inflammation-related genes in epididymal adipose tissues. Values are means ±S.E.M. (n = 6). *P<0.05.

It is known that the inflammation of adipose tissues is a cause of obesity-induced insulin resistance and glucose intolerance [32], [33]. Therefore, we studied inflammation in epididymal adipose tissues of ahKO mice. Immunofluorescence measurements showed that F4/80, a marker for macrophage, was increased in HFD WT mice compared with ND WT mice (Fig. 2A). It is known that the macrophages infiltrating the adipose tissues formed CLS around adipocytes. In ahKO mice, the obesity-induced CLS formation was decreased, indicating that macrophage infiltration is decreased in ahKO mice (Fig. 2A and 2B). In addition, F4/80 mRNA levels in the adipose tissues were increased in HFD WT mice compared with ND WT mice, whereas the HFD-induced increase of F4/80 was attenuated in ahKO mice (Fig. 2C). This result also suggests that the knockout of HIF-1α in adipocytes suppresses the infiltration of macrophages into the adipose tissues. Moreover, the mRNA levels of TNFα and MCP-1, which are adipokines acting as pro-inflammatory mediators, were decreased in the adipose tissues of HFD ahKO mice (Fig. 2C). In serum, MCP-1 levels were not decreased in HFD ahKO mice compared with HFD WT mice (Fig. S2), suggesting that the local expression of MCP-1 is an initiator of obesity-induced inflammation. Together, the results indicated that obesity-induced macrophage infiltration and inflammation in adipose tissues were reduced in ahKO mice.

**Figure 3:**
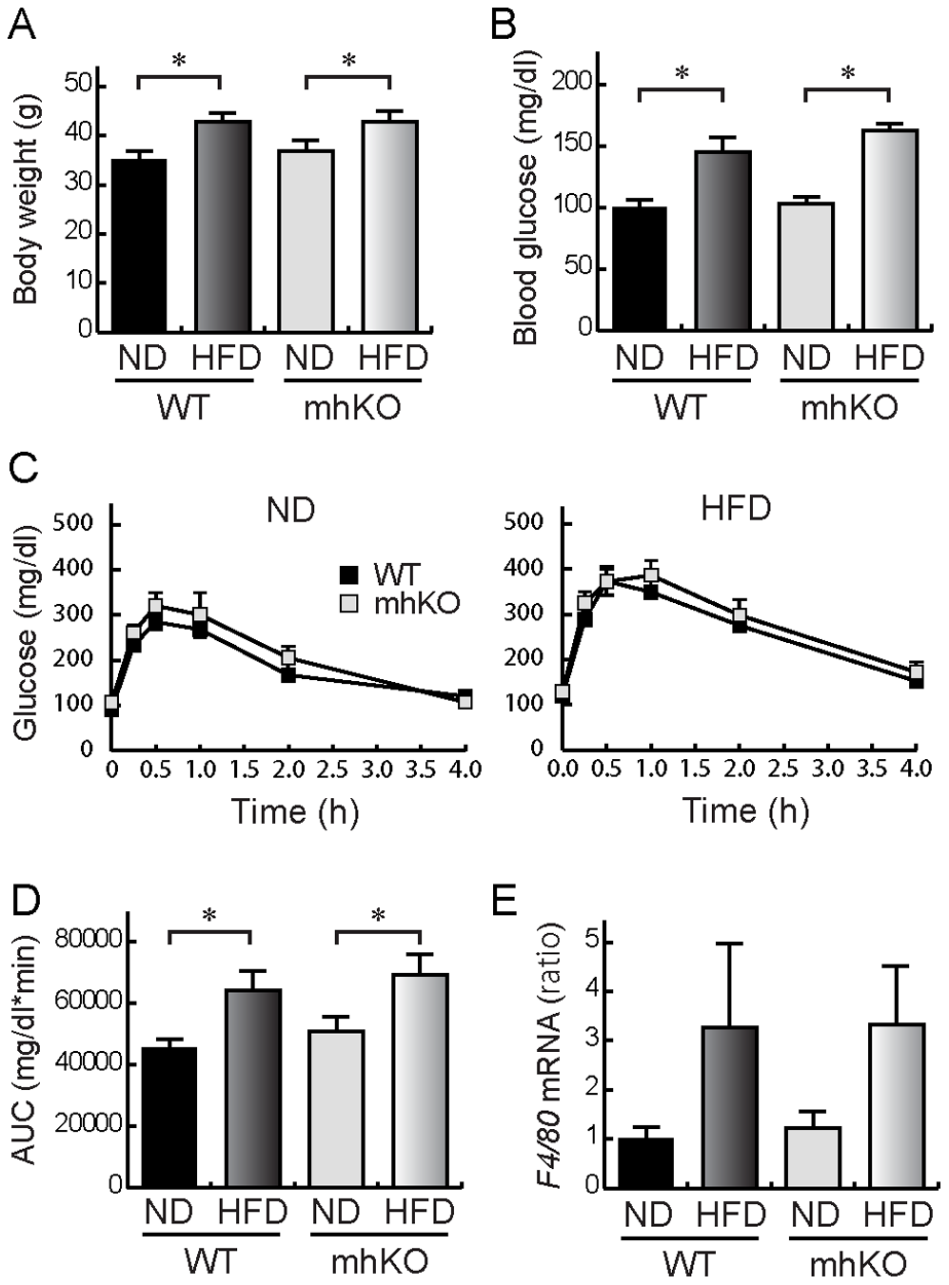
Characteristics of mhKO mice. (A) Body weights of WT and mhKO mice at the age of 15 weeks. Values are means ±S.E.M. (n = 6). *P<0.01. (B) Blood glucose levels after a 24-h fast. Values are means ±S.E.M. (n = 9∼10). *P<0.01. (C) Time-dependent changes of blood glucose concentrations in OGTT. Values are means ±S.E.M. (n = 9∼10). (D) AUCs of glucose concentration in OGTT. Values are means ±S.E.M. (n = 9∼10). *P<0.01. (E) mRNA levels of F4/80 in epididymal fat pads of mhKO mice are shown. mRNA levels were determined with real-time PCR. Values are means ±S.E.M. (n = 6).

In the present study, knockout of HIF-1α was accomplished by using mice expressing Cre recombinase under the control of the aP2 promoter. However, it is known that the promoter is active in macrophages [34]. Therefore, it is possible that the reduction of macrophage infiltration and inflammation in ahKO mice is due to the knockout of HIF-1α in macrophages. To rule out this possibility, we prepared macrophage-specific HIF-1α knockout (mhKO) mice. As shown in Fig. 3A, mhKO mice gained weight under both ND and HFD conditions, and the increase was comparable to that observed in WT mice. Moreover, the HFD-induced increase of blood glucose level was not different between the mice, indicating that obesity was induced in both mhKO mice and WT mice (Fig. 3B). In contrast to ahKO mice, there were no significant differences in the time courses of blood glucose levels between WT mice and mhKO mice (Fig. 3C and 3D). In addition, the obesity-induced increase of F4/80 mRNA levels in epididymal adipose tissues was not reduced in mhKO mice (Fig. 3E), indicating that macrophage infiltration in obese epididymal fat was not decreased in mhKO mice. The results strongly supported that the deletion of HIF-1α in adipocytes, but not in macrophages, contributed to the improvement of obesity-induced adipose tissue inflammation and glucose tolerance in ahKO mice.

### Increase of serum insulin levels in ahKO mice

**Figure 4:**
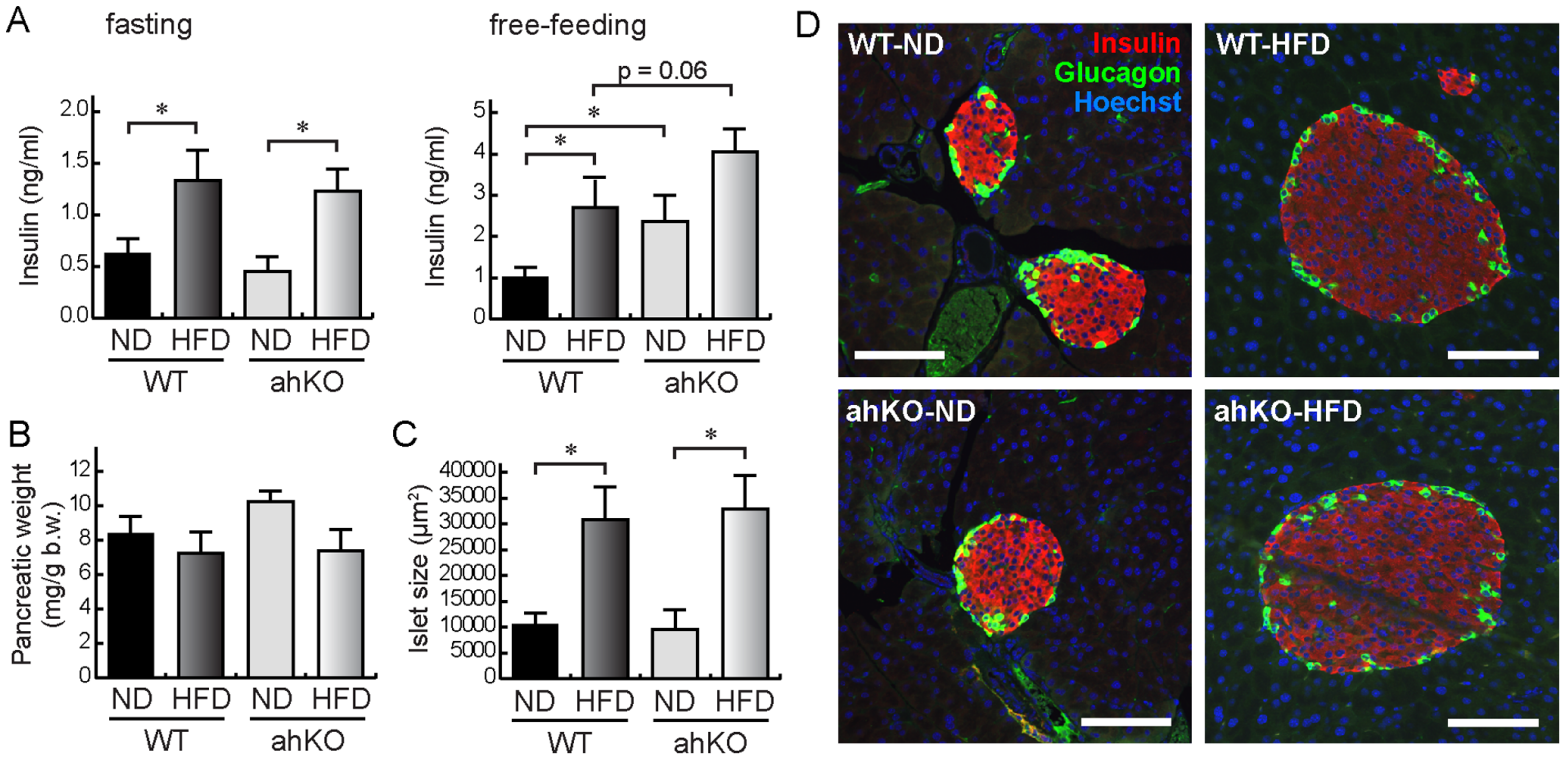
Serum insulin concentrations and pancreatic characteristics of ahKO mice. (A) Serum insulin levels under free-feeding and fasting conditions. Values are means ±S.E.M. (n = 6). *P<0.01. (B) Pancreatic weights. Values are means ±S.E.M. (n = 6). (C) Sizes of pancreatic islets. Values are means ±S.E.M. (n = 6). *P<0.01. (D) Immunostaining images of pancreatic islets. Immunostaining was performed with anti-insulin (red) and anti-glucagon (green) antibodies. Nuclei were counterstained with Hoechst (blue). Bars indicate 100 μm.

To further evaluate the characteristics of ahKO mice, we measured serum insulin levels. Under the fasting condition, serum insulin levels were increased in HFD WT mice compared with their ND counterparts (Fig. 4A). The ahKO mice exhibited identical serum insulin levels to WT mice under the fasting condition. However, under the free-feeding condition, the levels were significantly increased in ND ahKO mice compared with ND WT mice (Fig. 4A). In addition, the insulin levels under the free-feeding condition tended to increase in HFD ahKO mice compared with HFD WT mice (Fig. 4A). The results suggested that postprandial insulin secretion was stimulated, leading to the improvement of glucose tolerance in ahKO mice.

As shown in Fig. 4B, pancreatic weights were similar to one another. Immunostaining showed no differences in islet sizes and compositions of β- and α-cells between ahKO mice and WT mice (Fig. 4C and 4D). In addition, no differences were observed in the insulin fluorescence intensities between WT and ahKO mice. The results suggested that the increased insulin secretion was not due to pancreatic hyperactivity. Therefore, it is expected that incretin hormones, namely, GLP-1 and glucose-dependent insulinotropic polypeptide (GIP), are involved in the increase of insulin levels.

### Involvement of GLP-1 in improved glucose tolerance in ahKO mice

**Figure 5:**
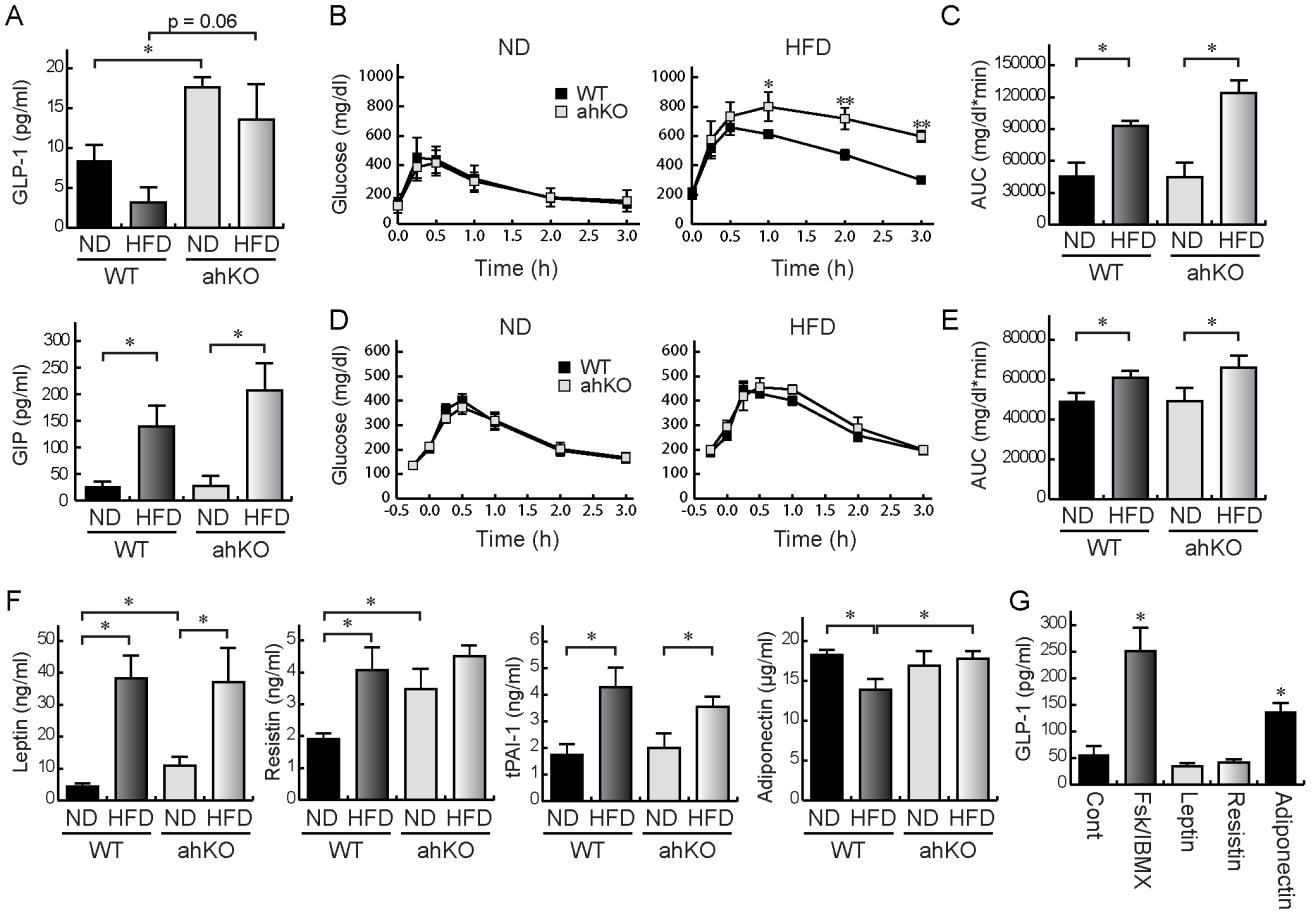
Involvement of GLP-1 in improvement of glucose tolerance in ahKO mice. (A) Serum GLP-1 and GIP levels under free-feeding condition. Values are means ±S.E.M. (n = 6). *P<0.01. (B) Time-dependent changes of blood glucose concentrations in ipGTT. After a 24-h fast, 2 g/kg glucose was injected intraperitoneally. Values are means ±S.E.M. (n = 5). *P<0.05 and **P<0.01. (C) AUCs of glucose concentrations in ipGTT. Values are means ±S.E.M. (n = 5). *P<0.01. (D) Time-dependent changes of blood glucose concentrations in OGTT with pre-injection of Ex9–39. Twenty five μg of Ex9–39 per kg body weight was injected. Fifteen minutes after Ex9–39 injection, 2 g/kg glucose was administered orally. Values are means ±S.E.M. (n = 5). (E) AUCs of glucose concentrations in OGTT with pre-injection of Ex9–39. Values are means ±S.E.M. (n = 5). *P<0.01. (F) Serum concentrations of leptin, resistin, tPAI-1, and adiponectin are shown. Values are means ±S.E.M. (n = 6). *P<0.01. (G) GLUTag cells were treated with 100 nM leptin, 10 ng/ml resistin, or 30 μg/ml adiponectin for 2 h and GLP-1 secreted into the incubation buffer was measured with ELISA. Ten uM Fsk/IBMX was used as positive control for GLP-1 secretion. Values are means ±S.E.M. (n = 5). *P<0.01.

Serum GLP-1 and GIP levels under the free-feeding condition were measured. Serum GLP-1 levels were markedly increased in ahKO mice under the ND condition and tended to increase under the HFD condition, whereas serum GIP levels were not different between WT mice and ahKO mice (Fig. 5A). To determine the involvement of GLP-1 in improving glucose tolerance in ahKO mice, we performed ipGTT, in which the intraperitoneal injection of glucose did not induce intestinal GLP-1 secretion, and OGTT with pre-injection of Ex9-39, an inhibitor of GLP-1 receptor. The ipGTT showed no improvement of glucose clearance in ahKO mice under both ND and HFD conditions. Glucose clearance in HFD ahKO mice worsened, although the increase of AUC showed no significant difference (Fig. 5B and 5C). Pre-injection of Ex9-39 abolished the improvement of glucose tolerance observed in ahKO mice (Fig. 5D and 5E). These observations indicated that GLP-1 contributes to the improvement of glucose tolerance in ahKO mice.

### Enhancement of GLP-1 secretion from L cells by adiponectin

We hypothesized that adipokines stimulated GLP-1 secretion from intestinal L cells. First, we determined changes in serum adipokine levels between WT and ahKO mice. MILLIPLEX MAP was used to detect leptin, tPAI-1, and resistin. In addition, adiponectin was detected with an ELISA kit. Although serum tPAI-1 levels were not different between WT mice and ahKO mice, serum leptin and resistin levels were increased in ND ahKO mice compared to ND WT mice (Fig. 5F). In addition, adiponectin was significantly increased in HFD ahKO mice compared with HFD WT mice (Fig. 5F). Then, the expression levels of adiponectin in epididymal adipose tissues were observed by western blot. The expression level of adiponectin was not increased in the adipose tissues, suggesting that another adipose tissue is the source of adiponectin (Fig. S3). Next, we studied whether the adipokines stimulated GLP-1 secretion from intestinal L cells. GLUTag cells, an intestinal L cell line, were treated with 100 nM leptin, 10 ng/ml resistin, or 30 μg/ml adiponectin for 2 h, and GLP-1 concentrations in the incubation buffer were measured. As shown in Fig. 5G, adiponectin stimulated GLP-1 secretion from the L cells. The result suggests that adiponectin is one of the factors for the secretion of GLP-1 from intestinal L cells.

## Discussion

In the present study, we examined the involvement of adipocyte HIF-1α in the development of obesity-induced diabetes in ahKO mice. The ahKO mice exhibited improved glucose tolerance compared with WT mice (Fig. 1). We found that serum insulin levels were markedly increased in ahKO mice under the free-feeding condition (Fig. 4). As the increased serum insulin levels were not observed under the fasting condition (Fig. 4) and ITT showed no insulin resistance in ahKO mice (Fig. 1), postprandial insulin secretion, but not compensatory hyperinsulinemia, was probably enhanced in ahKO mice, leading to the improved glucose tolerance.

There are two possibilities for the increased serum insulin levels. One is the enhancement of insulin secretion by pancreatic β-cells and the other is the effect of incretin hormones. Our results showed that the enhancement of insulin secretion is probably due to GLP-1, because no increases in islet size or insulin content and marked increases in serum GLP-1 levels were observed in ahKO mice (Figs. 4 and 5). ipGTT, which does not induce intestinal GLP-1 secretion, failed to improve glucose tolerance in ahKO mice (Fig. 5). In addition, in OGTT, the pre-injection of Ex9-39 abolished the improvement of glucose tolerance in ahKO mice (Fig. 5). These GTT results strongly support the involvement of GLP-1 in improving glucose tolerance in ahKO mice. In addition to directly stimulating insulin secretion, GLP-1 also helps to confer glucose sensitivity to pancreatic β-cells by stimulating glucose transporters and glucokinase [3], [4]. Moreover, GLP-1 amplifies insulin-mediated glucose uptake in adipocytes [35] and acutely increases microvascular recruitment and basal glucose uptake in muscle [36]. The pleiotropic effects of GLP-1 probably enhanced glucose tolerance in ahKO mice.

GLP-1 seems to have improved glucose tolerance in ahKO mice. However, it is not clear why the knockout of HIF-1α in adipocytes increases intestinal GLP-1 secretion. We found that adiponectin is capable of inducing the secretion of GLP-1 from GLUTag cells, an intestinal L cell line (Fig. 5). Therefore, it is possible that adipocyte-specific HIF-1α knockout increases adiponectin secretion from the adipocytes and the secreted adiponectin, in turn, induces GLP-1 secretion from intestinal L cells. To our knowledge, this is the first report of the effect of adiponectin on GLP-1 secretion. A previous study showed that the inhibition of HIF-1α increased the expression of adiponectin through the SOCS3-STAT3 pathway [37]. In ahKO mice, the disruption of HIF-1α in adipocytes may also increase adiponectin expression via the same pathway. It is known that exendin-4, a GLP-1 receptor agonist, induces adiponectin expression in adipocytes as well [38]. The reciprocal induction of adiponectin and GLP-1 probably participates in the regulation of whole-body glucose homeostasis. However, the source of adiponectin was not determined in the study because adiponectin protein levels were not increased in the epididymal and subcutaneous fat pads of the ahKO mice (Fig. S3). As adiponectin is expressed mainly in adipocytes, the source may be another adipose tissue, such as mesenteric and perirenal adipose tissues. Further studies are needed to reveal the source of adiponectin in the ahKO mice.

It is known that low-grade inflammation is evoked in obese adipose tissues, which is a cause of whole-body insulin resistance [32], [33]. Our study showed that macrophage infiltration was attenuated in adipose tissues of obese ahKO mice (Fig. 2). In addition, MCP-1 and TNFα mRNA expression was reduced (Fig. 2), indicating that obesity-induced inflammation is attenuated in ahKO mice. The deletion of HIF-1α in macrophages did not reduce F4/80 mRNA levels in epididymal fat pads (Fig. 3). Therefore, HIF-1α expressed in adipocytes probably triggers the macrophage infiltration into adipose tissues. It is known that MCP-1 promotes macrophage infiltration into adipose tissues [8]–[10]. It is also known that MCP-1 is directly regulated by HIF-1α in astrocytes [39] and adipocytes under the hypoxic condition exhibit increased expression of MCP-1 [40]. Therefore, HIF-1α probably regulates MCP-1 in adipocytes, leading to the attenuation of both macrophage infiltration and inflammation in adipose tissues of ahKO mice. The results indicated that the attenuation of inflammation improved insulin sensitivity and glucose tolerance in ahKO mice. It is reported that GLP-1 inhibits macrophage infiltration into adipose tissues [41]. The increased serum GLP-1 level is also perhaps an additional contributor to the attenuation of both macrophage infiltration and inflammation in adipose tissues of ahKO mice.

Jiang et al. studied the effects of HIF-1α expressed in adipocytes on the development of diabetes by using adipocyte-specific HIF-1α disrupted mice (Hif1α^ΔAdipo^) [28]. Their study revealed decreased body weight and fat mass; reduced adipocyte size; improved insulin resistance and glucose tolerance; inhibition of macrophage infiltration into adipose tissues, and increased serum adiponectin levels in Hif1α^ΔAdipo^ mice compared with WT mice. Our results also showed improved insulin resistance and glucose tolerance (Fig. 1), inhibition of macrophage infiltration into adipose tissues (Fig. 2), and significant increases in serum adiponectin levels in HFD ahKO mice (Fig. 5). However, our results showed no differences in body weight, fat mass, and adipocyte size between WT and ahKO mice. As a recent study has demonstrated the uncoupling of adiposity and insulin resistance [42], HIF-1α probably has no direct effect on adiposity. In addition to the report of Jiang et al., our results provide important aspects of the effect of HIF-1α on the induction of obesity-induced diabetes, namely, the involvement of GLP-1 in improving glucose tolerance in ahKO mice and the involvement of HIF-1α expressed in adipocytes, but not in macrophages, in the initiation of obesity-induced macrophage infiltration into adipocytes, as described above.

Insulin resistance and the failure of insulin secretion from pancreatic β-cells are particularly critical for obesity-induced type 2 diabetes [43], [44]. The deletion of HIF-1α in adipocytes stimulated postprandial insulin secretion through the adiponectin-GLP-1 pathway. In addition, ahKO mice showed improved insulin sensitivity. Our results suggest that one reason for the improved insulin sensitivity is the reduction of macrophage infiltration and inflammation through the decrease of MCP-1. Therefore, the deletion of HIF-1α in adipocytes improved glucose tolerance via two distinct pathways: decrease of insulin resistance and improvement of insulin secretion.

## Supporting Information

Figure S1 **Expression levels of HIF family.** (A) mRNA levels of HIF-1α in epididymal fat, liver, and muscle. Real-time PCR was performed with cDNA produced from total RNA isolated from the tissues. Values are means ±S.E.M. (n = 6). †P<0.01 vs. ND WT mice and ‡P<0.01 vs. HFD WT mice. (B) Western blot images of HIF family. Epididymal fat pads isolated from ahKO and WT mice were solubilized in lysis buffer and subjected to western blotting. (C) Quantification of protein levels of HIF. Values are means ±S.E.M. (n = 6). *P<0.01.
(TIF)

Figure S2 **Serum concentration of MCP-1.** Serum concentration of MCP-1 was measured with a MILLIPLEX MAP Mouse Adipokine Magnetic Bead Panel. Values are means ±S.E.M. (n = 8). *P<0.01.
(TIF)

Figure S3 **Adiponectin expression in adipose tissues.** Adiponectin expression levels in epididymal adipose tissues (A) and subcutaneous adipose tissues (B) were estimated by western blotting. Values are means ±S.E.M. (n = 5∼7). *P<0.05. Ponceau indicates the strongest band with Ponceau staining.
(TIF)
