# Supplementary figures and images for: Deletion of Hypoxia-Inducible Factor-1α in Adipocytes Enhances Glucagon-Like Peptide-1 Secretion and Reduces Adipose Tissue Inflammation
Source: PLoS One. 2014 Apr 4;9(4):e93856. doi: 10.1371/journal.pone.0093856 (PMC3976326; doi:10.1371/journal.pone.0093856)

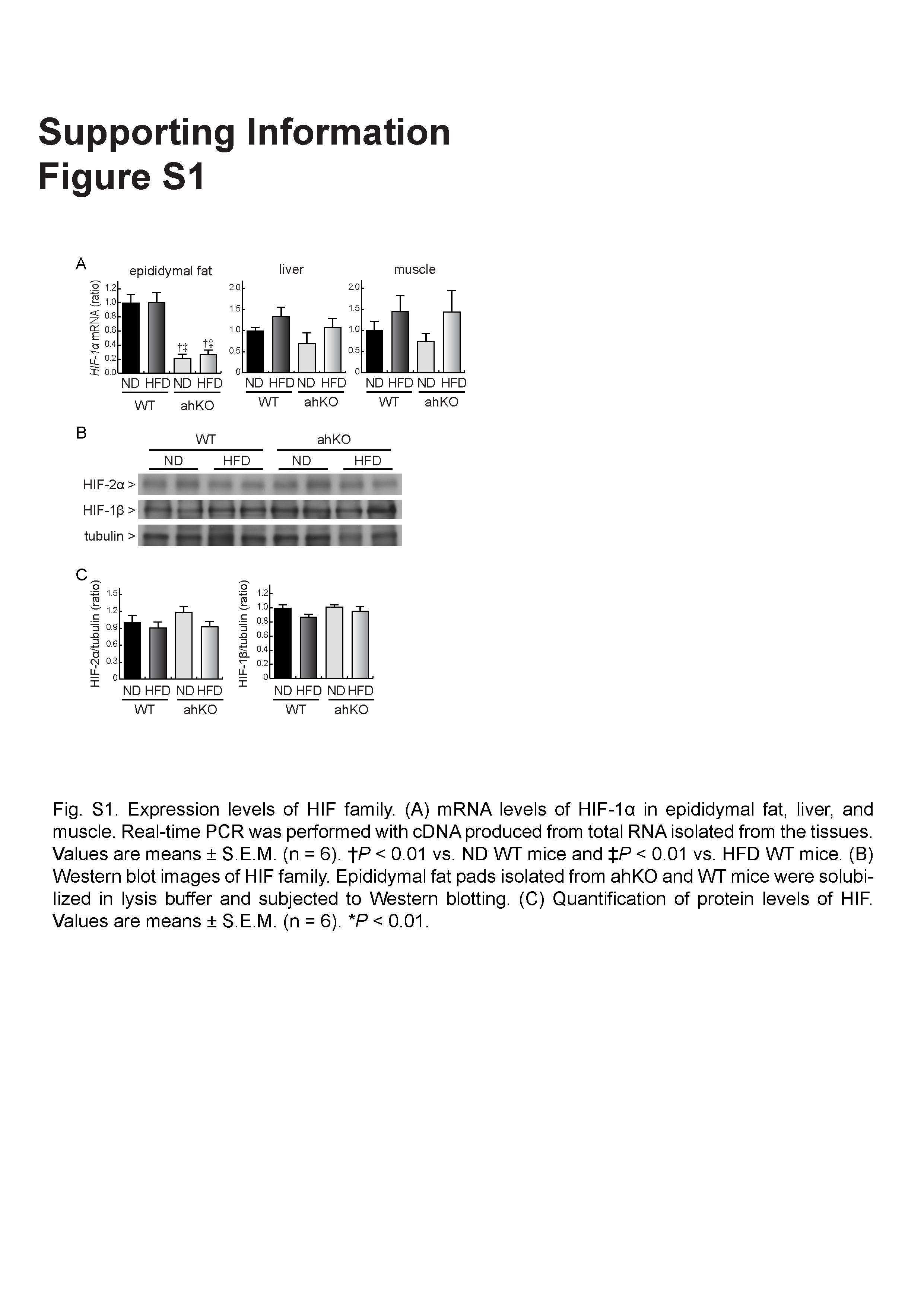

Supplement: Figure S1 — Expression levels of HIF family. (A) mRNA levels of HIF-1α in epididymal fat, liver, and muscle. Real-time PCR was performed with cDNA produced from total RNA isolated from the tissues. Values are means ±S.E.M. (n = 6). †P<0.01 vs. ND WT mice and ‡P<0.01 vs. HFD WT mice. (B) Western blot images of HIF family. Epididymal fat pads isolated from ahKO and WT mice were solubilized in lysis buffer and subjected to western blotting. (C) Quantification of protein levels of HIF. Values are means ±S.E.M. (n = 6). *P<0.01. (TIF) [file pone.0093856.s001.tif]

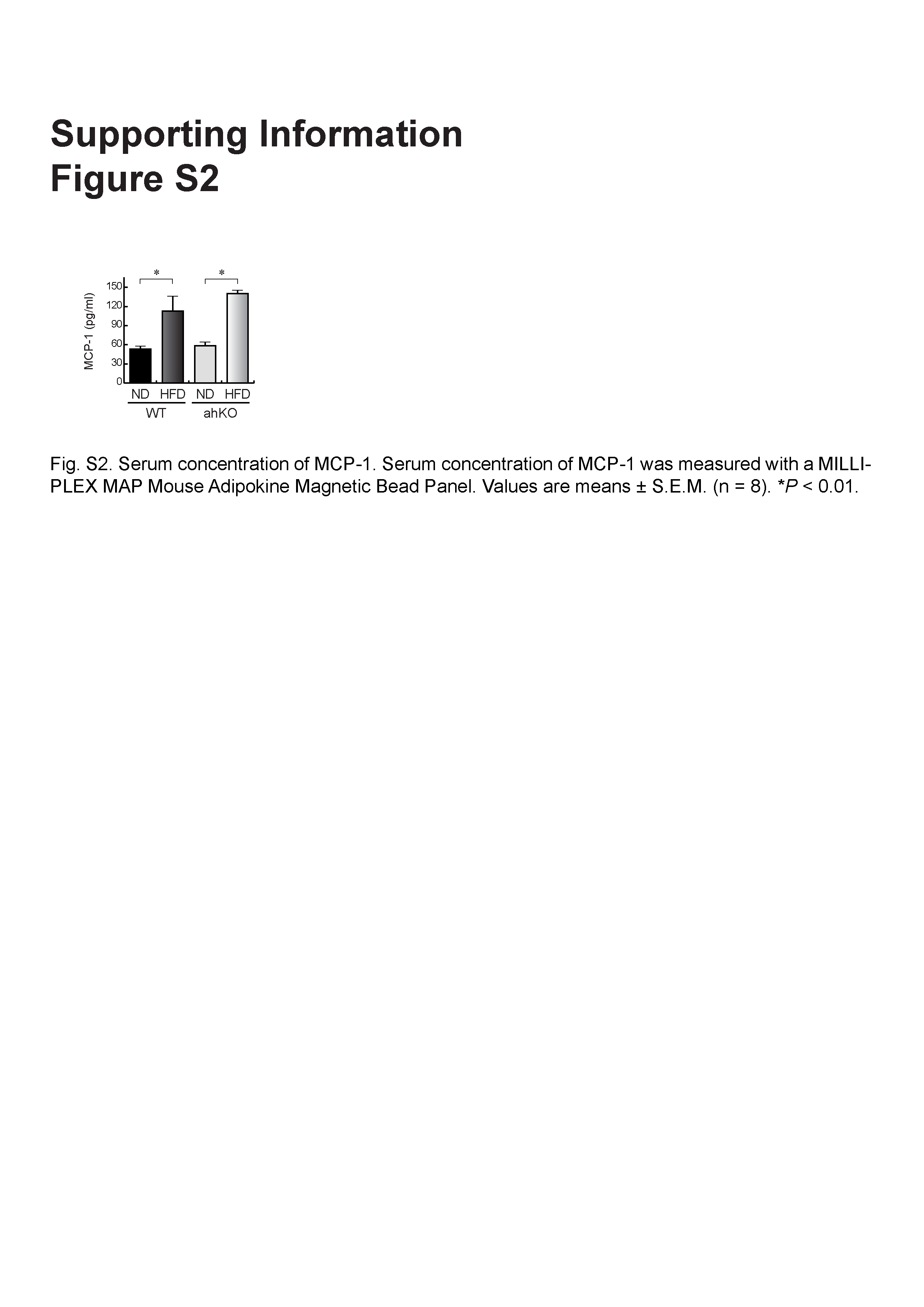

Supplement: Figure S2 — Serum concentration of MCP-1. Serum concentration of MCP-1 was measured with a MILLIPLEX MAP Mouse Adipokine Magnetic Bead Panel. Values are means ±S.E.M. (n = 8). *P<0.01. (TIF) [file pone.0093856.s002.tif]

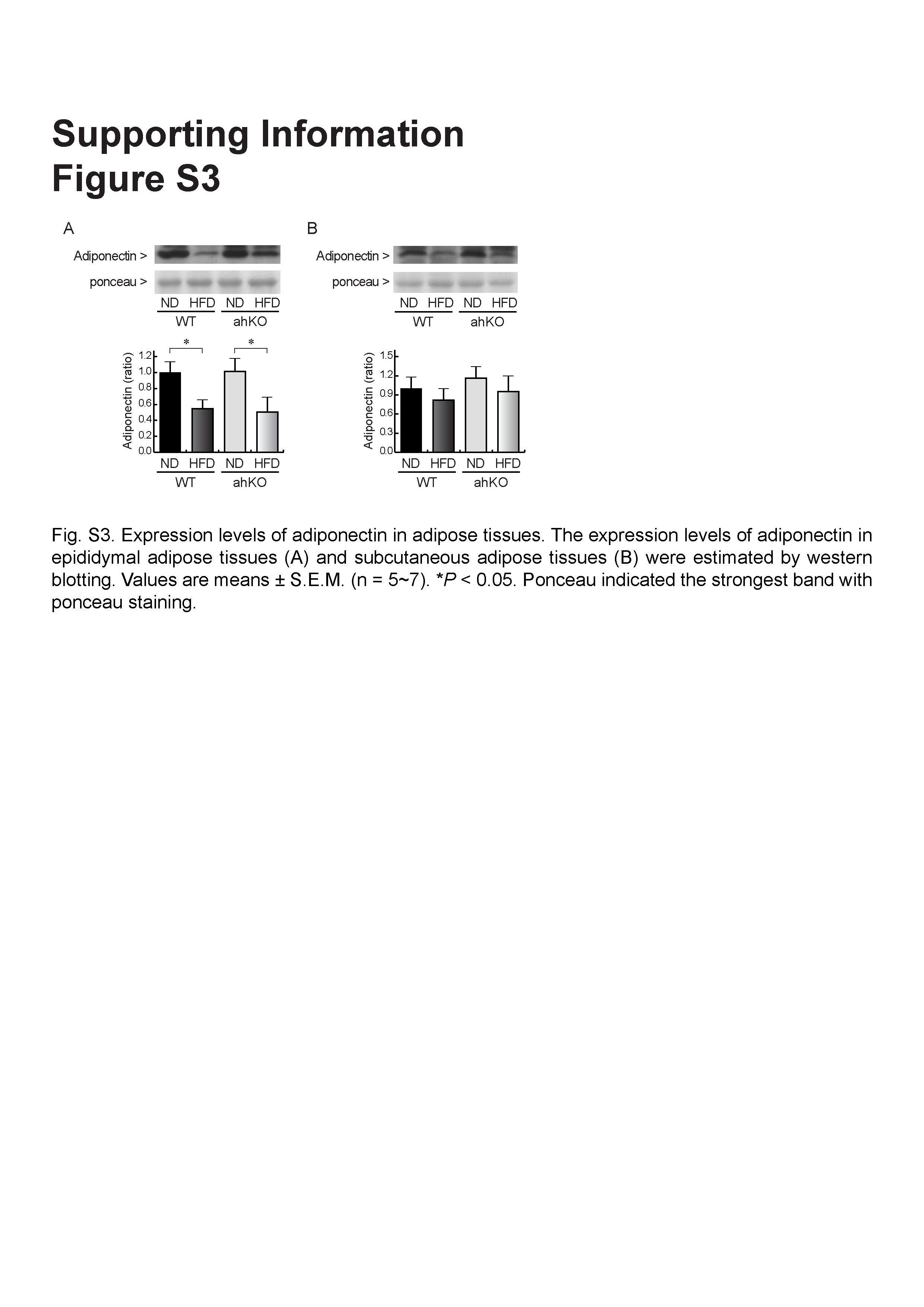

Supplement: Figure S3 — Adiponectin expression in adipose tissues. Adiponectin expression levels in epididymal adipose tissues (A) and subcutaneous adipose tissues (B) were estimated by western blotting. Values are means ±S.E.M. (n = 5∼7). *P<0.05. Ponceau indicates the strongest band with Ponceau staining. (TIF) [file pone.0093856.s003.tif]
